# Differential Temperature Effects on Phenolic Recovery and Antioxidant Capacity During the Hydroethanolic Extraction of Chilean Blueberry Pomace

**DOI:** 10.3390/antiox15070883

**Published:** 2026-07-16

**Authors:** Franco Espinoza-Cartagena, Manuel Benítez-Fajardo, Sebastián Ormazábal-Latorre, Laura M. Cuellar, Camilo Sánchez-Baeza, Gonzalo A. Núñez, Andrés F. Arroyo-Avirama

**Affiliations:** 1 Center for Systems Biotechnology (CSB), Universidad Andrés Bello, Fernández Concha 700, Santiago 7591538, Chile; 2 Department of Chemical and Environmental Engineering, Universidad Técnica Federico Santa María, Av. Vicuña Mackenna 3939, Santiago 8940897, Chile; 3 Departamento de Ingeniería Química y Bioprocesos, Escuela de Ingeniería, Pontificia Universidad Católica de Chile, Avenida Vicuña Mackenna 4860, Santiago 7820436, Chile; 4 Centro Interdisciplinario de Investigación Biomédica e Ingeniería para la Salud (MEDING), Universidad de Valparaíso, General Cruz 222, Valparaíso 2340003, Chile; 5 Doctorado en Biotecnología, Facultad de Ciencias de la Vida, Universidad Andrés Bello, Avenida República 440, Santiago 8370186, Chile; 6 Escuela de Ingeniería Bioquímica, Pontificia Universidad Católica de Valparaíso, Av. Brasil 2085, Valparaíso 2340025, Chile

**Keywords:** blueberry pomace, *Vaccinium corymbosum*, anthocyanins, antioxidant capacity, FRAP, DPPH

## Abstract

Blueberry pomace is a polyphenol-rich by-product of fruit processing. A face-centered central composite design (FCCCD) optimized hydroethanolic extraction across time (20–60 min), temperature (20–60 °C) and ethanol (20–80%), evaluating total phenolic content (TPC), yield and selectivity. The selected condition (40 min, 60 °C, 50% ethanol) reached 20.0 mg GAE/g dry biomass, a yield of 27% and a selectivity of 75.2 mg GAE/g dry extract. Peleg’s model adequately described the kinetics at 20 and 60 °C; pseudo-equilibrium was reached within 5 min, with equilibrium concentration rising from 9.9 to 20.8 mg GAE/g DW, and initial rate tripling. Based on the kinetic analysis (t_99_ ≈ 2.5 min), 5 min was adopted as the operational reference for phytochemical characterization; extracts were characterized by FRAP, DPPH, total anthocyanin content (TAC), and HPLC-DAD anthocyanin profiling. Increasing the temperature from 20 to 60 °C raised FRAP 3.4-fold and TAC 1.9-fold while reducing DPPH by 14%, decoupling bulk phenolic recovery from antioxidant capacity. HPLC-DAD identified five glycosylated anthocyanins, with petunidin-3-glucoside as the dominant constituent. Antimicrobial screening confirmed activity against *Staphylococcus aureus*. These results provide foundational process and compositional data to guide GRAS-compatible valorization of Chilean blueberry pomace and caution against bulk phenolic indicators as the sole quality criteria.

## 1. Introduction

Chile is one of the principal global producers of blueberries (*Vaccinium corymbosum*), with recent national outputs exceeding 130,000 tons annually [1,2]. While most of this volume targets the fresh export market, increasing regional competition has diverted a growing fraction, historically around 9 to 15%, toward juice and concentrate processing [3]. This industrial activity generates significant solid residues, primarily skins, seeds, and pulp remnants, which account for 20 to 30% of the input fruit weight [4,5]. As a result, the Chilean industry produces thousands of tons of blueberry waste each year. Despite its high content of anthocyanins, phenolic acids, and dietary fiber [6,7], over 80% of this biomass is currently discarded in landfills or composted [4], representing a substantial loss of bioactive resources and an environmental burden.

Blueberry pomace, composed mainly of skins, seeds, and pulp remnants, retains a phytochemical profile that in several respects surpasses that of the fresh fruit. Total phenolic contents of up to 22.33 mg GAE/g DW and total flavonoid contents of 19.41 to 21.65 mg/g DW have been reported, with the latter significantly exceeding values measured in whole blueberries [4,8]. Ethanolic extraction further concentrates this bioactive fraction, yielding extractives with two- to threefold higher levels of phenolics, flavonols, and anthocyanins relative to the unextracted pomace [9]. Anthocyanins constitute the dominant polyphenol class, with at least 13 glycosylated derivatives of five aglycone bases (malvidin, delphinidin, petunidin, cyanidin, and peonidin) identified by HPLC-DAD and LC-MS/MS, although relative abundances vary markedly among cultivars and geographic origins [6,7,9,10]. The phenolic acid fraction is dominated by hydroxycinnamic acid derivatives, while flavanols and B-type procyanidins represent additional polyphenol families of relevance [4,6]. Dietary fiber accounts for 15 to 25% of pomace dry weight, with the soluble fraction contributing up to 69% of the fiber-associated antioxidant capacity [4,5]. The functional potential of this composition has been validated through in vitro antioxidant assays (DPPH, FRAP, ABTS) across multiple extraction technologies [6,8,11], while enzyme inhibition studies have demonstrated notable α-glucosidase and α-amylase inhibitory activities, suggesting anti-diabetic potential [12,13]. Furthermore, polyphenol-rich blueberry extracts have shown antimicrobial functionality when incorporated into active packaging, reducing disease incidence in stored fruit by up to 33% [14]. These properties have been translated into diverse product applications, including fortified dairy products [15], bakery formulations with enhanced fiber content and phenolic bioaccessibility [16], and anthocyanin-based intelligent packaging films with antioxidant and UV-barrier functionality [17,18].

The recovery of polyphenols from blueberry pomace has been pursued primarily through non-conventional technologies such as ultrasound-assisted extraction (UAE), pulsed electric fields (PEF), microwave-assisted extraction, and Natural Deep Eutectic Solvents (NADESs), often optimized via response surface methodology (RSM) [10,19,20,21]. By contrast, conventional hydroethanolic extraction has received limited optimization effort for this matrix, despite this technique being the most industrially accessible and GRAS-compliant approach. RSM-optimized conventional extraction has proven effective for other fruit pomaces [22,23], and Troncoso Mesa et al. [24] applied a Box–Behnken design to optimize SLE of Chilean *V. corymbosum* cv. Legacy pomace achieved a TPC of 335.95 mg GAE/100 g, a total anthocyanin content of 272.69 mg Cyn-3-glu/100 g, and a DPPH-based antioxidant capacity of 528.96 mg TE/100 g. However, that study relied exclusively on spectrophotometric determinations (Folin–Ciocalteu, pH-differential, and DPPH), without reporting individual anthocyanin profiling using HPLC or complementary antioxidant assays that capture different radical scavenging and reducing mechanisms [4,25]. Since anthocyanin profiles and phenolic composition are strongly cultivar- and environment-dependent [26], and since individual anthocyanin identity governs specific bioactivities including antimicrobial efficacy [14], a comprehensive characterization of Chilean blueberry pomace extracts remains a significant gap. The combined optimization of SLE via RSM with kinetic modeling of the extraction process and an extended analytical panel encompassing TPC, HPLC-based anthocyanin profiling, a double antioxidant assessment (DPPH, FRAP), and antimicrobial activity evaluation has not been reported for this matrix. Since FRAP and DPPH differ fundamentally in their reaction mechanisms, electron transfer under acidic conditions versus hydrogen-atom and electron transfer toward a sterically hindered radical, temperature-driven shifts in the composition of the recovered phenolic fraction may dissociate these two metrics in ways that a single-assay approach cannot detect [27,28].

The aim of this work was to optimize the conventional hydroethanolic extraction of phenolic compounds from Chilean blueberry (*Vaccinium corymbosum*) pomace and to deliver a comprehensive characterization of the resulting extracts that addresses the gaps identified above. A face-centered central composite design (FCCCD) was applied to evaluate the effects of extraction time, temperature and ethanol concentration on total phenolic content, total extraction yield and selectivity. The selected condition was independently validated at 20 and 60 °C, and the extraction kinetics were modeled with Peleg’s model and a pseudo-first-order model to identify a conservative operational time point. The extracts obtained at that time point were then characterized by HPLC-DAD anthocyanin profiling, with two complementary antioxidant assays (FRAP and DPPH) targeting different reaction mechanisms; total anthocyanin content was assessed by the pH-differential method; and antimicrobial activity was determined against *Staphylococcus aureus* and *Escherichia coli*. By combining response surface optimization, kinetic modeling, individual anthocyanin profiling and a complementary functional bioactivity panel, this study provides a more complete temperature-dependent description of Chilean blueberry pomace extracts than previously available and supports their valorization as a food-grade source of bioactive phenolic compounds. The scope of the present contribution is deliberately restricted to the optimization of the extraction process and to the chemical and in vitro antioxidant characterization of the resulting extracts. Cell-based functional assays (intracellular ROS attenuation and cytocompatibility) and in vivo studies, which would require dedicated experimental designs including cell-line selection, oxidative-stress models and dose–response evaluation, are explicitly framed as future work and are not part of the present contribution. The in vitro antioxidant indicators reported here (FRAP, DPPH, total phenolic and anthocyanin content, and antimicrobial assays) correspond to internationally accepted methods of primary characterization of plant-derived extracts and are consistent with the level of evidence required for studies focused on process optimization and chemical fingerprinting [29].

## 2. Materials and Methods

### 2.1. Chemical Reagents

The following analytical-grade reagents were purchased from Sigma-Aldrich (St. Louis, MO, USA): Folin–Ciocalteu reagent, sodium carbonate, anhydrous sodium acetate, potassium chloride, ferric chloride (FeCl_3_), TPTZ (2,4,6-tris(2-pyridyl)-s-triazine), Trolox (6-hydroxy-2,5,7,8-tetramethylchroman-2-carboxylic acid), DPPH (2,2-diphenyl-1-picrylhydrazyl), gallic acid, and ampicillin. Cyanidin-3-glucoside was provided by Phytolab (Vestenbergsgreuth, Germany), whereas delphinidin-3-galactoside, cyanidin-3-galactoside, petunidin-3-glucoside, peonidin-3-galactoside, and malvidin-3-galactoside were supplied by Biosynth (Staad, Switzerland). Hydrochloric acid was obtained from Merck (Darmstadt, Germany), and ethanol was purchased from Winkler (Santiago, Chile). Methanol and formic acid (both HPLC grade) were acquired from Supelco (Bellefonte, PA, USA). Mueller–Hinton agar was purchased from Becton Dickinson (Franklin Lakes, NJ, USA). Distilled water was produced in-house using a water distillation system (model 10DESA0041, Pobel, Madrid, Spain). All other reagents and solvents used were of analytical or HPLC grade, as appropriate.

### 2.2. Raw Material Preparation

Dried and ground blueberry pomace (*Vaccinium corymbosum*, primarily of the Brigitta and Legacy cultivars) was obtained from a local industrial producer of blueberry vinegar located in Temuco (Araucanía, Chile), where it was generated as the solid residue from the pressing of whole blueberries prior to acetic fermentation of the resulting juice. The material was sieved, and the fraction smaller than 355 μm was separated and frozen at −20 °C until use.

### 2.3. Experimental Design

The effect of time, temperature, and ethanol concentration in aqueous solutions on the extraction yield of phenolic compounds was studied using a face-centered central composite design (FCCCD). The response variables used were TPC, total extraction yield, and selectivity. Three factors were applied, where 15 experimental runs (the central point included) were performed in triplicate. The following quadratic polynomial model (Equation (1)) was fitted to the experimental data:

(1) $$y=\beta_{0}+\sum_{i=1}^{N}\beta_{i}X_{i}+\sum_{i=1}^{N}\sum_{j=1}^{N}\beta_{ij}X_{i}X_{j}+\sum_{i=1}^{N}\beta_{ii}X_{i}^{2}$$

where y corresponds to responses, $\beta_{0}$ is the intercept, and $\beta_{i},\beta_{ij}$ and $\beta_{ii}$, are the linear, interactive, and quadratic terms, respectively. The $X_{i}$ and $X_{j}$ terms correspond to coded factors, which take on values of −1, 1, or 0, as shown in Table 1.

### 2.4. Extraction Procedure

Aqueous ethanol mixtures were chosen as the solvent system because ethanol is GRAS. This enables future uses of the extracts in food applications. Hence, 1 g of sieved blueberry pomace was weighed and mixed with 20 mL of previously heated aqueous ethanol mixtures (20, 50, or 80%) in a 50 mL flask. The system was maintained under shaking at 200 rpm for the required time (20, 40, or 60) and at the required temperature (20, 40, or 60 °C), according to the experimental design (Table 1). The extracts were centrifuged at 4000 rpm for 15 min using a centrifuge 5810R (Eppendorf, Hamburg, Germany). The supernatant was recovered for further analysis, while the remaining wet solid was dried at 105 °C for 24 h, until constant weight was obtained. All extracts were analyzed on the day of preparation, with the exception of those used for antimicrobial assays, which were stored at 4 °C in the dark for a maximum of 24 h prior to use.

### 2.5. Determination of Extraction Yield

The overall extraction yield (Y) was determined gravimetrically by calculating the mass difference in the pomace before the extraction process and after drying, expressed as a percentage of the initial pomace dry mass.

### 2.6. Analysis of Total Phenolic Content (TPC)

TPC was determined using the Folin–Ciocalteu method described by Singleton et al. [30]. Briefly, 0.1 mL of extract was mixed with 0.5 mL of Folin–Ciocalteu reagent and 4.9 mL of water in a volumetric flask. After 5 min, 1.7 mL of 20% sodium carbonate was added, and the flask was immediately shaken; then, the volume was adjusted with distilled water to 10 mL. The mixture was maintained in the dark for 2 h, and the absorbance was measured in a spectrophotometer (Infinite M200 Pro, Tecan, Switzerland) at 760 nm using the extraction solvent as a blank. The total phenolic content was determined using a standard curve and expressed as milligrams of gallic acid equivalents per gram of dry weight of pomace (mg GAE/g DW).

### 2.7. Determination of Selectivity

Extraction selectivity (S) was calculated as the ratio of total phenolic content to total extraction yield (Equation (2)):

(2) $$S=\frac{TPC\;\left(\frac{mg\;GAE}{g\;DW}\right)\times 100}{Y\;\left(\%\right)}\;$$

where S was expressed as the total phenols per gram of dry extract (mg GAE/g DE). This metric quantifies the phenolic enrichment of the dry extract relative to the total extracted mass, providing an operational index of extraction selectivity. It should be noted that, since TPC is determined by the Folin–Ciocalteu method, the metric reflects the concentration of Folin-reactive compounds in the extract and does not independently discriminate phenolics from other co-extracted matrix components such as free sugars or organic acids.

### 2.8. Kinetic Modeling

The kinetic behavior of TPC was evaluated at different extraction times (0, 0.5, 2, 5, 10, 20, 40 and 60 min). Two experimental conditions were selected: the optimal condition obtained from the FCCCD, and a second condition at the same aqueous ethanol concentration but at the lowest temperature evaluated (20 °C).

The temporal evolution of the extracted TPC (C(t)) was fitted to two empirical models: Peleg’s model (Equation (3)) and a pseudo-first-order model (Equation (4)). These models were chosen as they have been widely applied in the literature to describe the extraction of bioactive compounds from plant matrices [31,32].

(3) $$C\left(t\right)=\frac{t}{k_{1}+k_{2}t}\;$$

(4) $$C\left(t\right)=C_{\infty }\left(1-e^{-kt}\right)$$

where $k_{1}$ is the Peleg constant rate associated with the initial extraction rate, $k_{2}$ is related to the extraction capacity, $C_{\infty }$ represents the pseudo-equilibrium concentration, and k is the apparent first-order rate constant.

The time required to reach 99% of the pseudo-equilibrium concentration ($t_{99}$) predicted by Peleg’s model is given by Equation (5):

(5) $$t_{99}=99\times \frac{k_{1}}{k_{2}}$$

This parameter is used as the operational reference for the kinetic characterization of the extraction process.

### 2.9. Phytochemical and Biological Evaluation of Extracts Obtained at 5 min

#### 2.9.1. HPLC Analysis of Anthocyanins

Anthocyanins were characterized using HPLC for extracts produced at 20 °C and 60 °C, both with an extraction time of 5 min.

Samples were filtered using a 0.45 μm membrane filter before analysis. The analysis was carried out using a Dionex Ultimate 3000 HPLC system equipped with a diode array detector (DAD) (Thermo Fisher Scientific, Waltham, MA, USA). Detection was performed at 520 nm, which is suitable for anthocyanin identification.

Separation was achieved using a C18 column (Symmetry C18, Waters) of 5 μm, which was 4.6 mm × 150 mm under gradient elution conditions. The separation protocol entailed a mobile phase comprising 5% formic acid in water (solvent A) and 100% methanol (solvent B). Prior to analysis, all solvents underwent filtration through a 0.45 μm membrane. The gradient program for the system was as follows: 0–5 min, 10–15% B; 5–15 min, 15–20% B; 15–20 min, 20–25% B; 20–25 min, 25–30% B; 25–45 min, 30–60% B; and finally, 3 min, 100% B. A constant flow rate of 1.0 mL/min was maintained.

Individual anthocyanins were identified by comparing retention times with those of analytical standards (delphinidin-3-galactoside, cyanidin-3-galactoside, cyanidin-3-glucoside, petunidin-3-glucoside, peonidin-3-galactoside, and malvidin-3-galactoside) analyzed under identical chromatographic conditions. Chromatographic data were acquired and processed using Chromeleon software version 7.2.8 (Thermo Fisher Scientific, Waltham, MA, USA). Quantification was performed by external calibration using cyanidin-3-glucoside as a reference standard over the concentration range of 0.005–0.41 mg/mL. Although cyanidin-3-glucoside was not detected in the blueberry pomace extracts, its use as a universal reference is consistent with established analytical practice in berry anthocyanin analysis, where spectral similarities among anthocyanins make single-standard quantification feasible and cyanidin-3-glucoside equivalents represent the most widely adopted reporting convention [33,34]. Results were expressed as milligrams of cyanidin-3-glucoside equivalents per 100 g of dry blueberry pomace (mg Cyn-3-glu E/100 g DW). Individual anthocyanins were tentatively identified by co-elution with authentic commercial standards and by matching their UV-Vis absorption maxima ($\lambda_{max}$ in the 520–530 nm range characteristic of glycosylated anthocyanidins) under identical chromatographic conditions. This identification approach, based on retention times and UV-Vis spectral comparison with authentic standards, is widely adopted in food chemistry and has been recently applied in studies of berry and pomace matrices published in journals of comparable scope [6,35,36]. Mass-spectrometric confirmation by LC-MS/MS, which would constitute the most rigorous level of structural elucidation, was not within the analytical scope of the present study and is acknowledged as a methodological limitation in Section 3.4.1.

#### 2.9.2. Total Anthocyanin Content (TAC)

The total anthocyanin content was quantified using the pH-differential method, which exploits the structural transformation of anthocyanins under acidic conditions and relies on absorbance readings at 1.0 and 4.5 pH. Crude extracts were independently diluted in a 0.025 M hydrochloric acid–potassium chloride buffer (pH 1.0) and a 0.4 M sodium acetate buffer (pH 4.5). Optical measurements were performed at wavelengths of 500 and 700 nm using a microplate reader (Infinite M200 Pro, Tecan, Switzerland). The results were expressed as milligrams of cyanidin-3-glucoside equivalents per gram of dry weight (mg Cyn-3-glu E/g DW).

#### 2.9.3. Antioxidant Capacity Determination: FRAP Assay

Antioxidant capacity was evaluated using the FRAP assay described by Benzie and Devaki [37], with some modifications. Briefly, the FRAP reagent was prepared by mixing 300 mM sodium acetate buffer solution, 10 mM TPTZ, and 20 mM ferric chloride in volumetric proportions of 10:1:1. Mixtures prepared with 2.7 mL of the FRAP reagent and 0.3 mL of the sample were left for 30 min at 37 °C. Then absorbance was measured at 593 nm. A calibration curve was employed using Trolox as the standard; hence, the antioxidant capacity was expressed in micromoles of Trolox equivalent per gram of dry weight. (μmol TE/g DW).

#### 2.9.4. Antioxidant Capacity Determination: DPPH Assay

The DPPH radical scavenging assay was performed according to the methodology described in [38]. Briefly, 30 μL of either the diluted extract or Trolox standard was mixed with 150 μL of a 200 DPPH methanolic solution. The mixture was incubated in the dark at room temperature for 15 min, after which the absorbance was recorded at 517 nm. Antioxidant activity was quantified using a Trolox calibration curve and expressed as micromoles of Trolox equivalents per gram of dry substrate (μmol TE/g DW). All analyses were performed in triplicate.

#### 2.9.5. Antimicrobial Activity Assay

The antimicrobial activity of blueberry pomace extracts obtained at 20 °C and 60 °C was evaluated against *Staphylococcus aureus* and *Escherichia coli* using agar diffusion assays, including both disk diffusion and well diffusion methods, following previously described methodologies for the evaluation of bioactive compounds from natural sources [39,40].

Briefly, the bacterial inoculum was adjusted to a turbidity equivalent to 0.5 McFarland and uniformly spread onto Mueller–Hinton agar plates in three directions using sterile swabs. For the disk diffusion assay, sterile paper disks were impregnated with 15 μL of each extract solution, whereas in the well diffusion assay, 40 μL of extract solution at the same concentration was added to wells made in the agar. The extract solutions were concentrated to 263.5 mg DE/mL.

Ampicillin (10 μL, 5 mg/mL) was used as a positive control, while sterile water served as a negative control. All experiments were performed in triplicate. Plates were incubated at 37 °C for 24 h, and antimicrobial activity was determined by measuring the diameter of inhibition zones (cm) using image analysis (ImageJ v1.54g). Results were expressed as mean ± standard deviation.

### 2.10. Statistical Analysis

Experimental responses are reported as the mean ± standard deviation obtained from triplicate experiments. The 95% confidence interval was calculated for experimental responses obtained in FCCCD. The significance of statistical model coefficients (Table A1, Table A2 and Table A3) was evaluated by analysis of variance (ANOVA). The reproducibility of the selected condition was further evaluated by an independent one-way ANOVA between 20 and 60 °C for TPC, total extraction yield and selectivity at 40 min (n=5 per temperature; Table A4). Root mean square error (RMSE) was employed to evaluate and compare the kinetic models. All statistical analyses and model fitting were performed using Python (version 3.13) with the libraries NumPy (v2.4.6), SciPy (v1.17.1), pandas (v3.0.3) and statsmodels (v0.13.4). Pairwise comparisons of TPC, yield, selectivity, FRAP, DPPH, TAC, and inhibition zone diameters between methods and extraction temperatures were performed using Welch’s *t*-test, which does not assume equality of variances. A significance threshold of α=0.05 was applied in all tests (Table A5 and Table A6). Multiple-testing correction was performed within each a priori family of comparisons (six responses at the 5 min characterization and two methods in the antimicrobial assay) using the Benjamini–Hochberg false discovery rate (BH–FDR) procedure; reported significance codes refer to the adjusted p-values ($p_{\mathrm{adj}}$). No statistical test was applied to the *Escherichia coli* arm of the antimicrobial assay because all inhibition-zone observations were zero, leaving no within-group variance against which to test.

## 3. Results and Discussion

### 3.1. Experimental Responses Across the FCCCD Domain

**Figure 1:**
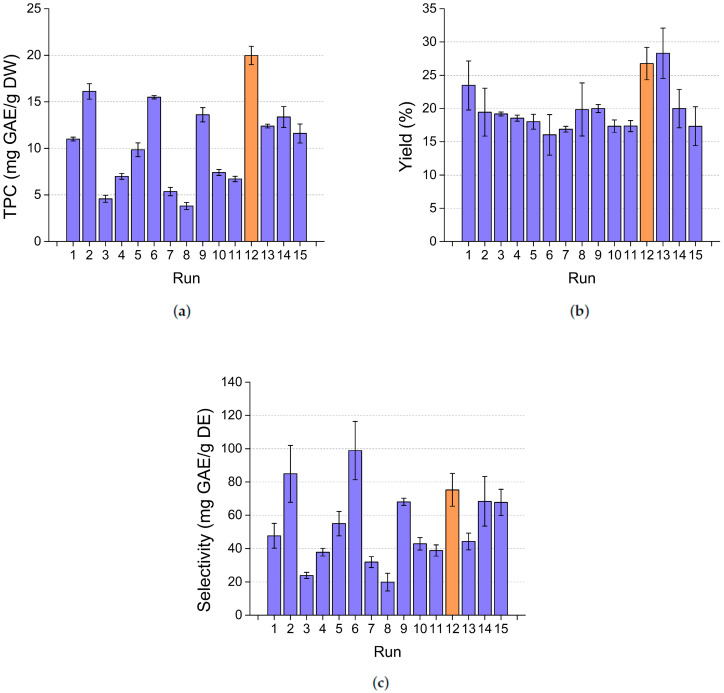
Experimental responses obtained across the face-centered central composite design (FCCCD). Bars represent mean values, and error bars indicate a 95% confidence interval for each experimental run: (**a**) total phenolic content (TPC), (**b**) total extraction yield, and (**c**) extract selectivity. Run numbers correspond to the experimental conditions listed in Table 2. The orange bar highlights run 12, which was selected as the reference condition for the subsequent analyses.

The experimental results obtained in triplicate using the FCCCD are presented in Table 2 and Figure 1. The response variables evaluated were TPC, overall extraction yield, and extract selectivity. TPC ranged from 3.81 to 19.97 mg GAE/g DW, while total yield ranged from 16.0 to 28.3%, and selectivity ranged from 19.9 to 98.77 mg GAE/g DE, indicating a wide variation in response within the studied conditions [41,42]. In particular, the highest TPC was obtained in run 12 (40 min, 60 °C, and 50% ethanol), with a value of 19.97 ± 0.98 mg GAE/g DW, while the highest yield was observed in run 13 (40 min, 20 °C, and 50% ethanol), at 28.3 ± 3.8%. Meanwhile, the highest selectivity was recorded in run 6 (20 min, 60 °C, and 20% ethanol), reaching 98.77 ± 17.46 mg GAE/g DW. These results show that there is no single condition that simultaneously maximizes all responses, which is consistent with findings from waste valorization processes, where overall yield and extract quality (phenolic enrichment) respond differently to operating variables and introduce a trade-off that must be considered when selecting the operating condition [43,44].

From a phenomenological perspective, conditions involving high temperatures tended to result in higher TPC values. This behavior has been widely documented in the literature, where it has been observed that temperature increases the solubility of phenolic compounds, reduces solvent viscosity, and improves mass transfer coefficients, promoting the release of compounds bound to the plant matrix [45,46]. In fact, several studies report that in conventional extractions, the maximum TPC is typically reached in the range of 60–80 °C, which is consistent with the results obtained in this study [45,47]. In contrast, overall yield showed a lower dependence on temperature, suggesting that total mass extraction is not necessarily governed by the same mechanisms as the selective recovery of phenolic compounds. This decoupling between yield and phenolic content has been observed previously, where increased temperature favors the extraction of specific compounds without necessarily increasing the total mass extracted [48,49]. Likewise, selectivity exhibited greater experimental dispersion, which is to be expected due to the propagation of errors since it is a variable derived from two independent measurements.

The statistical interpretation of the fitted models supports these experimental observations. For TPC, the quadratic model was highly significant (p<0.001), with high explanatory power ($R^{2}=0.962$; ${R^{2}}_{adj}=0.952$). Temperature exhibited the strongest positive linear effect (β=3.92; p<0.001), indicating that this variable is primarily responsible for the variation in response. This behavior is consistent with RSM optimization studies, where temperature typically emerges as the dominant factor in the extraction of phenolic compounds, surpassing the influence of time or solvent composition [41,50]. On the other hand, both time and ethanol concentration exhibited significant quadratic effects, indicating the existence of an optimum within the experimental range. This nonlinear response is characteristic of solid–liquid extraction systems, where an excessive increase in time or concentration can lead to solvent saturation or the co-extraction of undesirable compounds [43]. In particular, the negative quadratic effect of ethanol concentration (β=−6.26; p<0.001) suggests that intermediate solvent compositions maximize the recovery of phenolic compounds, a finding that has been widely reported for hydroalcoholic systems, where the polarity of the solvent determines the selective solubility of different types of compounds [48]. Although the quadratic models for TPC and selectivity displayed a significant lack of fit (Table A1 and Table A3), the direction and relative magnitude of the regression coefficients are used here only as descriptive indicators of factor importance; quantitative predictions and numerical optimization based on these models are therefore not warranted, and the operating condition was selected by direct comparison of the experimental responses, as discussed in the following section. The detection of a significant lack of fit in the TPC and selectivity models is in itself an informative outcome, indicating that the response surface within the experimental domain exhibits curvature of an order higher than two, consistent with the nonlinear behavior of solid–liquid extraction systems in which saturation, matrix-driven mass-transfer limitations and competing degradation reactions operate simultaneously [51]. Under these circumstances, the FCCCD nevertheless retained substantial practical value as a screening tool: it provided a structured exploration of the three operating variables, allowed the identification of statistically significant first-order and interaction effects, and offered a transparent dataset from which an operating condition could be selected by direct experimental observation rather than by model-based maximization. The independent runs reported in Table A4 (Appendix A) correspond to a reproducibility assessment of the selected operating condition (n = 5 per temperature) and not to a prediction-error evaluation against the RSM models, given that no model-based optimum was used.

For overall yield, the model showed lower explained capacity ($R^{2}=0.719$; ${R^{2}}_{adj}=0.646$), and temperature was not significant in its linear effect (p=0.835), confirming that this response is less sensitive to temperature. However, the quadratic terms were highly significant, suggesting that yield is governed by a balance between time, temperature, and solvent composition, rather than by a dominant linear effect. This type of behavior has been described in systems where multiple mechanisms (surface washing, internal diffusion, and solubilization) occur simultaneously [48,49]. In the case of selectivity, the model was also significant ($R^{2}=0.841$; ${R^{2}}_{adj}=0.800$), with a strong positive linear effect of temperature and negative effects associated with ethanol concentration. The significant interaction between temperature and solvent suggests that the thermal effect is coupled to the polarity of the extraction medium, which is consistent with reports where temperature modifies both the solubility and the profile of extracted compounds [45].

Taken together, these results indicate that temperature is the main driving factor in the extraction of phenolic compounds, while time and solvent composition define the optimal region through nonlinear effects. This trend has been consistently observed in processes for optimizing the extraction of bioactive compounds from agro-industrial by-products, reinforcing the consistency of the results obtained in this study [41,44]. These observations served as the basis for the selection of the optimal operating condition, as discussed in the following section.

### 3.2. Selection of the Operational Condition

The significant lack of fit observed for TPC (p≤0.001) and selectivity (p=0.010) models indicates that the fitted polynomial functions do not fully capture the response behavior within the experimental domain. Under these circumstances, using the model for numerical optimization is not statistically appropriate, since predictions derived from a poorly fitted surface may not reflect the true system response. Therefore, the selection of the best operating condition was carried out by direct comparison of the experimental results, supported by the 95% confidence intervals estimated for each run.

As described in Section 3.1, run 12 yielded the highest mean TPC, and its confidence interval did not overlap with those of most other runs, with the partial exception of run 2, the interval of which slightly overlaps with that of run 12. For total extraction yield, run 12 ranked second overall, behind run 13, although the confidence intervals of both runs overlap substantially, indicating no statistically significant difference between them for this response.

The selection of run 12 over other candidate conditions was guided by a functional and practical criterion. Runs with high selectivity values, such as run 2 and run 6, were obtained at conditions associated with lower overall yields (19.4 ± 3.6% and 16.0 ± 3.0%, respectively). Although the selectivity was higher in those cases, the lower mass recovery implies a reduced absolute amount of bioactive material obtained per gram of raw pomace. In contrast, run 12 combines the highest TPC with a yield above 25%, offering a more favorable balance between extract quality and total phenolic recovery.

The selected condition is also consistent with mechanistic reasoning. The combination of 60 °C and 50% ethanol promotes adequate solubility of phenolic compounds in the hydroalcoholic solvent while preserving selectivity, as intermediate ethanol concentrations are known to match the polarity of target compounds such as anthocyanins and hydroxycinnamic acid derivatives [49]. The intermediate extraction time of 40 min reflects the balance between a rapid initial phase driven by surface washing and a slower phase governed by internal diffusion, which is characteristic of solid–liquid extraction from plant matrices [52]. These considerations, together with the trends identified in Section 3.1 regarding the dominant role of temperature and the quadratic behavior of ethanol concentration, support run 12 as the most adequate operating condition within the evaluated experimental domain [41].

From the perspective of agro-industrial waste recovery, this condition also presents relevant practical advantages. Operating at 60 °C avoids the use of extreme conditions in temperature, which facilitates potential scale-up. Furthermore, the use of 50% aqueous ethanol is compatible with food-grade applications, as ethanol is classified as generally recognized as safe (GRAS) by the FDA, making the resulting extracts suitable for incorporation into functional foods or nutraceutical formulations.

**Figure 2:**
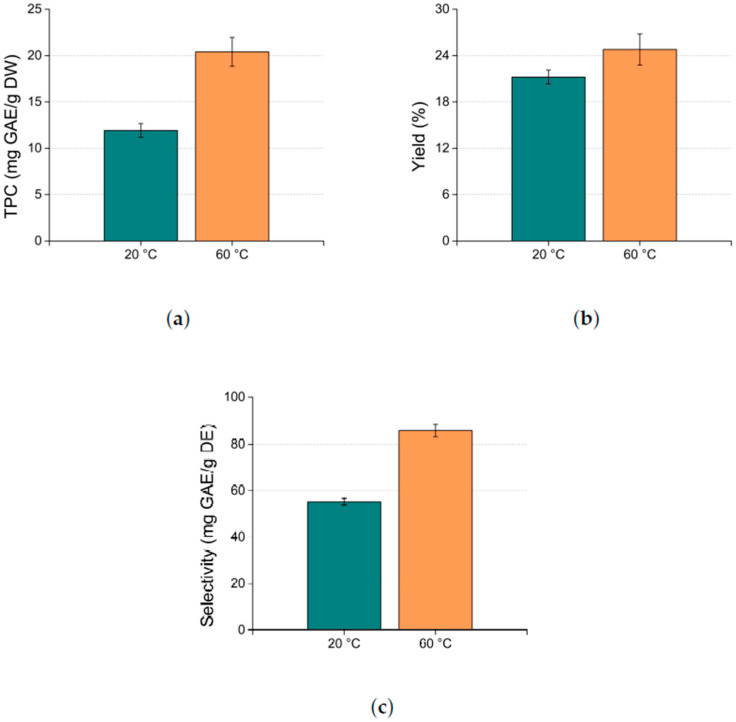
Validation of the optimal condition within the experimental domain with a neighboring condition obtained by decreasing the extraction temperature. Bars represent mean values and error bars indicate standard deviation (*n* = 5) for each experimental run: (**a**) total phenolic content (TPC), (**b**) total extraction yield, and (**c**) extraction selectivity.

To confirm the reproducibility of this condition, an independent validation experiment was performed with n = 5 replicates under the same operating conditions (40 min, 60 °C, 50% ethanol). Additionally, a neighboring condition at 20 °C was included to further assess the effect of temperature on extraction performance. The results are presented in Figure 2, and the corresponding one-way ANOVA between 20 and 60 °C for these three responses is summarized in Table A4 (Appendix A). The TPC, yield, and selectivity values obtained in the validation were consistent with those recorded in run 12 during the FCCCD, confirming the stability and reproducibility of the selected condition. The marked reduction in all three responses at 20 °C is in agreement with the statistical analysis presented in Section 3.1. This condition served as the reference point for the kinetic analysis that follows; however, the question of whether shorter extraction times could deliver an equivalent recovery, with substantial energy savings and lower thermal exposure, remained open and motivated the temporal evolution study presented next.

The TPC of 19.97 mg GAE/g DW achieved under the selected conditions compares favorably with values reported in the literature for hydroethanolic extraction of blueberry pomace and related matrices. Within the same matrix, the maximum TPC of 22.33 mg GAE/g DW reported by [8] was obtained by ultrasound-assisted extraction (UAE) under otherwise similar solvent conditions (50% ethanol), a technology requiring specialized equipment and additional energy input. By contrast, pulsed electric field (PEF) extraction in ethanol, another non-conventional approach, yielded only 10.52 mg GAE/g DW from blueberry pomace [6], and conventional solvent extraction (61 °C for 35 min at 70% ethanol) of *Vaccinium ashei* wine pomace achieved 5.08 mg GAE/g DW [19]. The value obtained in the present study thus represents a marked improvement over conventional benchmarks and approaches the performance of the UAE at substantially lower operational complexity. For context, conventional hydroethanolic extraction of grape pomace, a matrix more widely used industrially for polyphenol recovery, yields TPC values in the range of 17.91–35.10 mg GAE/g DW [53], within which the value reported here falls competitively. Taken together, these comparisons support the conclusion that RSM-optimized CSLE with aqueous ethanol constitutes an effective and scalable route for phenolic recovery from Chilean blueberry pomace.

### 3.3. Extraction Kinetics Modeling

**Figure 3:**
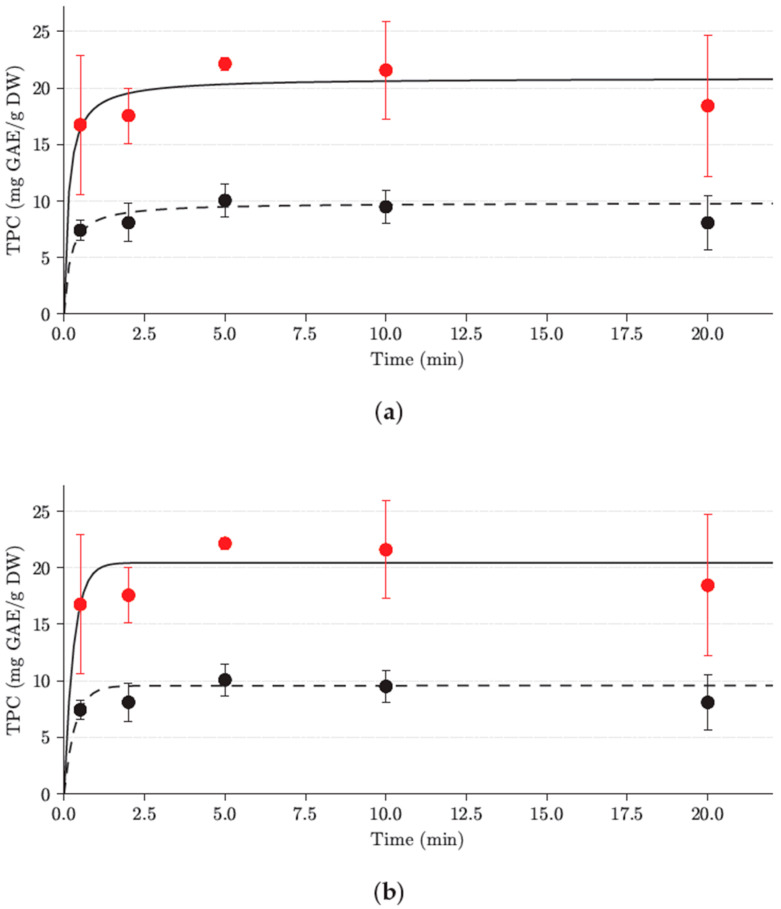
Extraction kinetics of total phenolic compounds (TPC) at 20 °C (•) and 60 °C (•). Continuous and dashed lines represent the fit for (**a**) Peleg’s model and (**b**) the first-order model, respectively. Experimental data are presented as the mean of three replicates (n=3), with error bars indicating the standard deviation. Although measurements were conducted up to 60 min, the time scale is limited to 20 min to provide a clearer visualization of the initial fast-extraction phase and model performance.

Polyphenol extraction kinetics were evaluated in terms of TPC at 0.5, 2, 5, 10, 20, 40, and 60 min (Figure 3). Temperature had a positive effect on the equilibrium concentration, with higher TPC values obtained at 60 °C than at 20 °C, which is consistent with the significant effect of temperature discussed in Section 3.1.

A rapid initial washing stage was observed, where compounds present mainly on the surface of the plant matrix were quickly solubilized, and pseudo-equilibrium was reached within the first 5 min. This behavior has been reported in mass-transfer-intensified systems such as UAE and MAE [54,55] and also in conventional solid–liquid extraction of phenolics from fruit-derived matrices [56]. The rapid attainment of pseudo-equilibrium is attributed to milling and selection of the <355 μm fraction, which increases the specific surface area and reduces intraparticle diffusion resistance.

Peleg’s model and first-order models were selected to represent the extraction kinetics due to their simplicity and effectiveness for bioactive compound extraction from plant matrices [31,32]. Both models predicted equilibrium concentrations in agreement with experimental plateau values (Table 3). For Peleg’s model, the equilibrium concentration was obtained as $C_{eq}=1/k_{2}$, yielding 9.9 and 20.8 mg GAE/g DW at 20 and 60 °C, respectively, which are close to the values predicted by the first-order model (9.56 and 20.44 mg GAE/g DW), confirming the consistency of both approaches.

Peleg’s model showed lower RMSE, MAE, and AIC at both temperatures (Table 3) and was therefore selected as the preferred model. However, since ΔAIC < 2 at both temperatures, both models provide statistically equivalent representations of the kinetic data, according to Burnham and Anderson’s selection criteria [57]. In addition to goodness-of-fit, Peleg’s model allows a more direct physical interpretation of the parameters: $k_{1}$ is inversely related to the initial extraction rate, and $k_{2}$ is inversely related to the equilibrium concentration. Both parameters decreased with increasing temperature ($k_{1}$ from 19.79 $\times 10^{-3}$ to 6.69 $\times 10^{-3}$ g mg GAE−1, and $k_{2}$ from 0.101 to 0.048 min g mg GAE−1), which is consistent with faster initial mass transfer and higher solute solubility at 60 °C. This separation between rate and equilibrium effects is not possible with the first-order model, where both are coupled through a single rate constant.

The experimental dispersion, reflected in RMSE values of 1.69 and 3.62 mg GAE/g DW at 20 and 60 °C, respectively, is attributed to the inherent heterogeneity of the pomace matrix. $R^{2}$ was not used as the primary goodness-of-fit metric, as it is not appropriate for nonlinear regression with plateau-type data [58].

No degradation of phenolic compounds was observed at either temperature, as TPC values remained stable after pseudo-equilibrium for up to 60 min. At 60 °C, thermal degradation of labile compounds such as anthocyanins could in principle be expected; however, the reported mean half-life of anthocyanins extracted using ethanol from blueberries (*Vaccinium corymbosum* L.) at 70 °C was 12.7 h [59], which implies a negligible degradation over the 60 min extraction window. This is consistent with the stable plateau observed experimentally and confirms that the selected conditions do not compromise the integrity of the extracted compounds.

The time required to reach 99% of the equilibrium concentration ($t_{99}$), estimated from Peleg’s model, was approximately 2.5 min. Identifying this parameter is relevant from a process engineering perspective, as it defines the minimum extraction time needed to achieve effective recovery, avoiding longer extractions that increase energy and solvent consumption without additional TPC gain. Therefore, although the FCCCD optimum and its validation were defined at 40 min, the kinetic evidence supports the fact that 5 min already captures the bulk of the extractable phenolic load. The 5 min time point is consequently adopted as the operational reference for the functional characterization of the extract presented in the following section, since it represents the most energy-efficient condition consistent with the kinetic plateau.

### 3.4. Phytochemical and Biological Characterization of Extracts at the Operational Time Point

**Figure 4:**
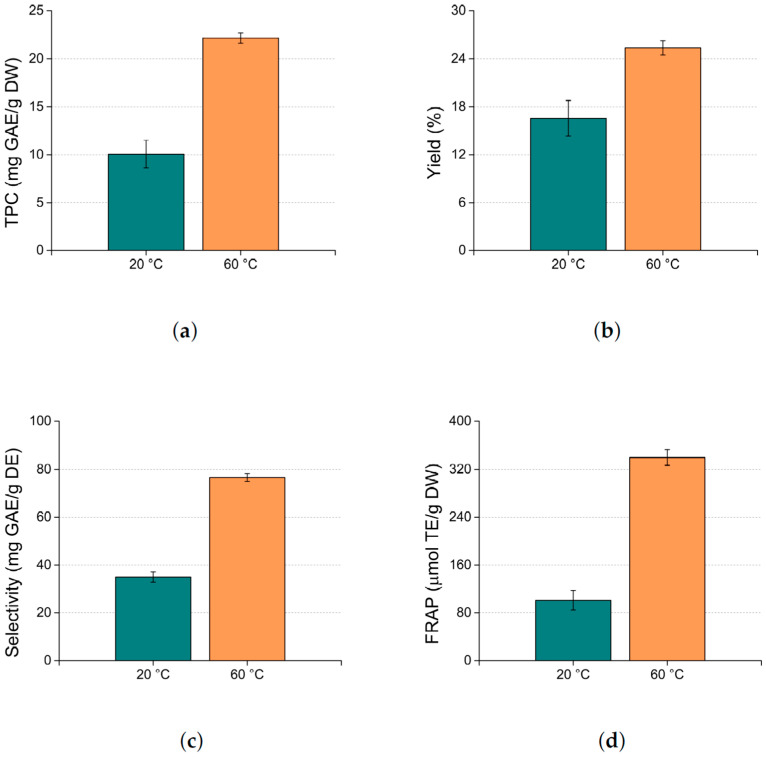

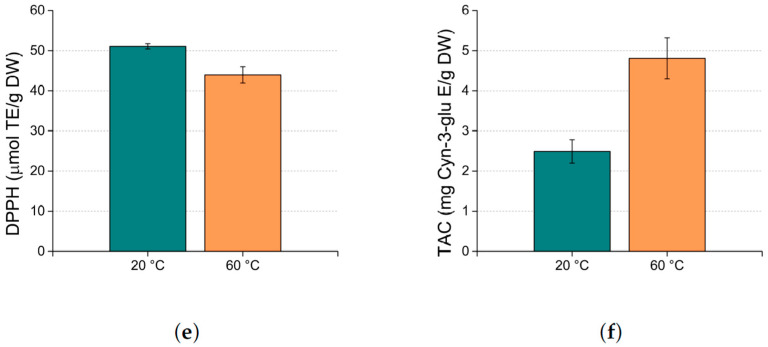
Characterization of the extracts obtained at 20 °C and 60 °C for 5 min: (**a**) total phenolic content (TPC), (**b**) total extraction yield, (**c**) extraction selectivity, (**d**) FRAP (ferric reducing antioxidant power), (**e**) DPPH radical scavenging activity, and (**f**) total anthocyanin content (TAC). Values are presented as means ± standard deviation (n=3).

Based on the kinetic analysis presented in Section 3.3, pseudo-equilibrium was attained within approximately 2.5 min at both temperatures, and the 5 min time point was selected as a conservative operational reference that ensures effective recovery while minimizing extraction time and solvent exposure. Extracts produced under these conditions at 20 °C and 60 °C were characterized in terms of anthocyanin profile, total anthocyanin content, antioxidant capacity, and antimicrobial activity, in order to assess the effect of extraction temperature on extract quality and bioactive potential. A global comparison of the responses obtained at 20 and 60 °C across the six measured variables (TPC, total yield, selectivity, FRAP, DPPH and TAC) is presented in Figure 4, and the corresponding two-tailed Welch’s t-test results are summarized in Table A5 (Appendix A).

#### 3.4.1. Anthocyanin Profile (HPLC-DAD)

The chromatographic profiles showed that both extracts were mainly composed of anthocyanins, with petunidin-3-glucoside and malvidin-3-galactoside as the dominant tentatively identified compounds. Other tentatively identified compounds included delphinidin-3-galactoside, cyanidin-3-galactoside, and peonidin-3-galactoside. The anthocyanin signature observed here, dominated by petunidin- and malvidin-glycosides with smaller contributions from delphinidin-, cyanidin-, and peonidin-glycosides, is consistent with the profile reported in the literature for the Brigitta and Legacy highbush blueberry cultivars [60,61].

A significant increase in peak areas was observed for each identified compound at 60 °C compared to 20 °C, indicating that temperature enhances the extractability of anthocyanins. For instance, the area of petunidin-3-glucoside increased more than sevenfold, consistent with the dominant effect of temperature on TPC recovery reported in Section 3.1.

Beyond total yield, temperature also modified the relative composition of the extracts (Table 4). At 20 °C, petunidin-3-glucoside represented approximately 50% of the total chromatographic area, while at 60 °C its contribution increased to 52%, and its absolute concentration increased from 29.23 to 96.24 mg Cyn-3-glu E/100 g DW. In contrast, the relative contribution of malvidin-3-galactoside decreased from 32% to 29%, whereas peonidin-3-galactoside, which remained below the detection limit at 20 °C, became quantifiable at 60 °C (8.59 mg Cyn-3-glu E/100 g DW). The emergence of this minor constituent only at the higher temperature is consistent with the markedly enhanced extraction of anthocyanins at 60 °C, which raised low-abundance compounds above the detection threshold of the method rather than reflecting any thermal generation of new species. Thermal degradation of the recovered anthocyanins is unlikely over the extraction window, since the reported mean half-life of anthocyanins in ethanolic blueberry extracts at 70 °C exceeds 12 h [59]. Identification of the five glycosylated anthocyanins reported here should be understood as tentative; definitive structural confirmation would require LC-MS/MS analysis, which falls beyond the analytical scope of the present study. The same approach (HPLC-DAD with authentic standards and UV-Vis spectra) has been recently used to characterize anthocyanin profiles of *Vaccinium myrtillus* pomace [36] and fifteen blackcurrant cultivars over a three-year period [35], supporting its validity as a primary identification tool in studies focused on extraction optimization and chemical characterization of berry-derived extracts.

These results indicate that temperature affects not only extraction yield but also the relative composition of the extract, which has implications for applications where specific anthocyanin identities govern bioactivity [14]. It should be noted that quantification of all anthocyanins was performed against a single external calibration of cyanidin-3-glucoside (Section 2.9.1), so concentrations are reported as cyanidin-3-glucoside equivalents and provide a robust within-compound comparison between the two extraction temperatures, but should be interpreted as semi-quantitative when compared across different anthocyanin identities, as individual response factors at 520 nm can differ slightly from those of cyanidin-3-glucoside.

It is important to acknowledge that the in vivo bioavailability of intact glycosylated anthocyanins is intrinsically low, with absorption rates typically reported below 2% in human studies, largely due to acid-driven structural transformations in the stomach, microbial metabolism in the colon and rapid plasma clearance [62,63]. The petunidin-3-glucoside-enriched profile observed at 60 °C should therefore be regarded as a chemical signature with potential for further development, rather than as a guarantee of bioactivity in vivo. Simulated gastrointestinal digestion models (INFOGEST-type protocols) and protective strategies based on encapsulation, complexation with food-grade carriers or co-formulation with absorption enhancers will be required to translate this profile into functional ingredient applications [64], and these dimensions constitute a natural extension of the present work.

#### 3.4.2. Total Anthocyanin Content and Antioxidant Capacity

The total anthocyanin content (TAC) of blueberry pomace obtained with aqueous ethanol was significantly higher (p<0.05) at 60 °C (4.81 ± 0.51 mg Cyn-3-glu E/g DW) than at 20 °C (2.49 ± 0.29 mg Cyn-3-glu E/g DW), representing an approximately 1.9-fold increase when tripling the extraction temperature (Figure 4f; Table A5, Appendix A). This result is consistent with the HPLC data presented in Section 3.4.1, where the absolute concentration of all identified anthocyanins increased markedly at 60 °C. The increase in extraction temperature from 20 to 60 °C led to a significant rise in total anthocyanin content (TAC) and ferric reducing antioxidant power (FRAP), whereas DPPH radical scavenging activity decreased (Figure 4d,e). This pattern suggests that increasing temperature promotes matrix disruption in blueberry pomace and enhances the release of phenolic compounds into the extraction medium, particularly anthocyanins, the recovery of which depends largely on mass-transfer efficiency and solubilization from the fruit skin, where they are predominantly associated with cell wall components such as pectins, hemicelluloses, and proteins [44,65]. In this context, the increase in TAC observed at 60 °C is consistent with previous reports on blueberry pomace extracts, in which temperature has been identified as a key variable affecting anthocyanin recovery [24].

The divergent response observed for FRAP and DPPH indicates that an increase in TAC does not necessarily translate into a comparable improvement in radical-scavenging activity against DPPH. This discrepancy can be explained by the different reaction mechanisms involved in both assays: FRAP evaluates the reducing capacity of an extract through electron transfer under acidic conditions, whereas DPPH relies on a mixed electron- and hydrogen-atom-transfer mechanism in which a bulky, sterically hindered radical is scavenged, making the assay more sensitive to the molecular structure and steric accessibility of the antioxidants present [27,28,29,66,67,68]. Thus, at 20 °C, a phenolic fraction with greater specific reactivity toward DPPH may be preferentially preserved or extracted, whereas at 60 °C, a broader recovery of compounds with higher overall reducing power is favored, as reflected by the increase in FRAP. The thermal degradation of the most labile anthocyanin species may also contribute to this kind of compositional shift [59,69]. While thermal degradation of anthocyanins to chalcone intermediates and phenolic acid derivatives, which are compounds that retain ferric-reducing capacity but exhibit lower radical-scavenging reactivity [59,69], represents a theoretically relevant pathway, the kinetic evidence presented in Section 3.3 ($t_{1/2}\gg$ extraction window) suggests that its contribution under the conditions of the present study is likely secondary to the differential mass-transfer effects described above.

This pattern is consistent with recent studies on berry by-products. Capaldi et al. [13] reported that blueberry pomace extracts with a higher proportion of monomeric polyphenols exhibited greater DPPH and ABTS activity than those with a higher total phenolic content but a larger proportion of polymeric tannins, suggesting that the degree of polyphenol polymerization negatively affects antioxidant activity assessed by hydrogen-transfer methods. Similarly, ref. [70] reported that the correlation between anthocyanins and DPPH activity can be only moderate in ethanolic berry pomace extracts, whereas the association with total polyphenol content is considerably stronger, indicating that phenolic compounds other than anthocyanins contribute substantially to the overall antioxidant response. Taken together, these findings support the idea that extraction temperature affects not only the total amount of compounds recovered, but also their qualitative composition and, consequently, their behavior in different antioxidant assays. It should also be noted that phenolic compounds other than anthocyanins, including hydroxycinnamic acid derivatives and flavonols, which are recognized components of blueberry pomace [4,6] but which were not characterized in the present study, may likewise contribute to the observed divergence in antioxidant capacity, and their differential extractability at 20 and 60 °C cannot be excluded.

From an applied perspective, the higher FRAP value at 60 °C suggests a more efficient recovery of compounds with global reducing capacity, whereas the higher DPPH response at 20 °C may be associated with a more selective fraction of antioxidants with greater reactivity toward this radical. Therefore, the combined assessment of TAC, DPPH, and FRAP provides a more complete characterization of the bioactive potential of the extracts, avoiding oversimplified interpretations based on a single antioxidant assay [27].

#### 3.4.3. Antimicrobial Activity

**Figure 5:**
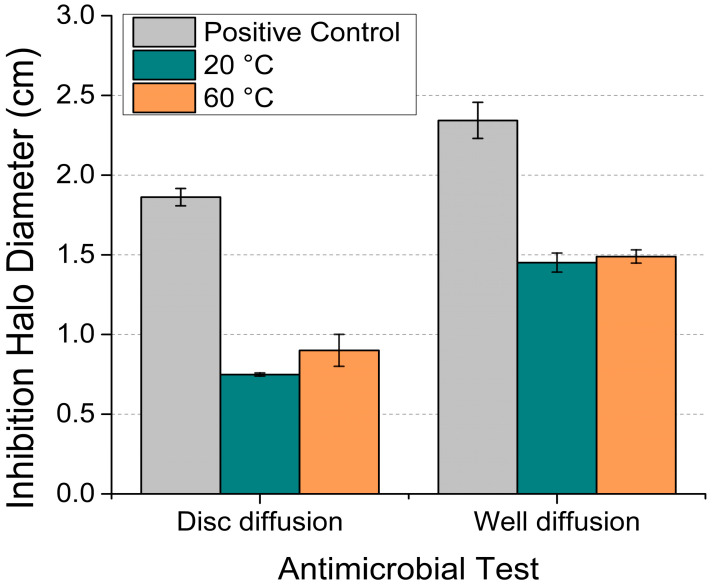
Antimicrobial activity of the extracts against *Staphylococcus aureus* (a Gram-positive bacterium) using two testing methods (disk diffusion and well diffusion).

The inhibition zone diameters obtained by disk and well diffusion for extracts produced at 20 °C and 60 °C, along with the positive control (ampicillin) and negative control (sterile distilled water), are summarized in Table 5 and shown in Figure 5.

No inhibition zone was detected for either extract against *Escherichia coli* under any tested condition. This lack of a detectable zone is consistent with the lower susceptibility of Gram-negative bacteria to anthocyanin-rich extracts [71,72], the outer membrane of which acts as a permeability barrier that restricts the diffusion of phenolic compounds into the cell [73]; under the concentration assayed, any inhibitory effect on *E. coli* therefore remained below the detection threshold of the diffusion method. The positive control (ampicillin) produced well-defined inhibition zones in both methods (disk: 1.861 ± 0.054 cm; well: 2.343 ± 0.113 cm), confirming strain susceptibility and assay validity. The negative control produced no measurable inhibition zone in any replicate. Both blueberry pomace extracts exhibited inhibitory activity against *Staphylococcus aureus* under all tested conditions, with halos that exceeded those of the negative control in every replicate. This contrasting susceptibility is mechanistically plausible: anthocyanins and related polyphenols are reported to interact preferentially with the peptidoglycan and lipoteichoic acid components of the Gram-positive cell wall, increasing membrane permeability and interfering with energy metabolism, whereas the lipopolysaccharide-rich outer membrane of Gram-negative bacteria restricts the access of these polar molecules to analogous targets [71,72]. In the present extracts, the marked enrichment of petunidin-3-glucoside at 60 °C (Section 3.4.1) did not translate into a measurable increase in inhibitory activity against *S. aureus*, indicating that the contribution of individual anthocyanins to antimicrobial efficacy is not proportional to their relative abundance in the recovered extract.

Well diffusion produced consistently larger inhibition zones than disk diffusion at both extraction temperatures, a difference that was statistically significant at 20 °C (t=−20.2, df=2.0, p<0.01) and at 60 °C (t=−9.4, df=2.7, p=0.01). This is consistent with the documented tendency of polyphenolic compounds to adsorb onto the hydrophilic surface of paper disks, reducing their effective diffusion into the agar, whereas liquid-filled wells avoid this limitation [74].

No statistically significant difference in inhibitory activity was observed between extracts obtained at 20 °C and 60 °C, either by disk diffusion (t=−2.6, df=2.0, p=0.12) or by well diffusion (t=−0.9, df=3.6, p=0.43; Table A6, Appendix A). Because both extracts were assayed at the same total concentration (263.5 mg/mL), the higher TPC, TAC, and FRAP values recovered at 60 °C did not translate into significantly greater inhibition of *S. aureus*, which is consistent with the view that the antibacterial activity of these extracts is not primarily driven by bulk phenolic content [75] but also depends on the identity of the phenolics recovered, consistent with the temperature-driven divergence already observed for the DPPH response [14]. In agar diffusion, zone size is set by the distance at which the diffusing extract falls to its minimum inhibitory concentration rather than by a linear dose response, so differences in loaded composition are expressed nonlinearly as the zone diameter [76]. This near equivalence should nevertheless be interpreted with caution, as the limited number of replicates (n=3) reduces the statistical power to detect a moderate temperature effect, and the modest change in relative anthocyanin composition between conditions (Section 3.4.1) is insufficient on its own to identify which constituents drive the observed activity. Confirming the compound-specific basis of this inhibition would require fractionation and compound-specific bioactivity assays.

Minimum inhibitory concentration (MIC) determination by broth microdilution was attempted for both extracts; however, reliable turbidimetric readings could not be obtained due to the intense coloration of the anthocyanin-rich extracts. This is a recognized limitation in antimicrobial susceptibility testing of polyphenol-rich matrices, and quantitative dose–response characterization would require alternative readout approaches [74].

## 4. Conclusions

The conventional solid–liquid extraction of phenolic compounds from blueberry pomace was successfully optimized by means of a face-centered central composite design using aqueous ethanol as a GRAS-compatible solvent. Extraction temperature was identified as the dominant factor governing the recovery, while extraction time and ethanol concentration showed significant quadratic effects, defining an optimum within the experimental domain at 40 min, 60 °C and 50% ethanol. Under these conditions, the process reached a total phenolic content of 19.97 ± 0.98 mg GAE/g DW, a total extraction yield of 26.8 ± 2.4% and a selectivity of 75.2 ± 9.85 mg GAE/g dry extract, and an independent one-way ANOVA confirmed that the reproducibility of this condition was statistically robust between 20 and 60 °C (Table A4, Appendix A). The kinetic study showed that pseudo-equilibrium was reached within the first 5 min at both temperatures, and the experimental data were adequately described by Peleg’s model. Increasing the temperature from 20 to 60 °C raised the estimated equilibrium concentration from 9.9 to 20.8 mg GAE/g DW and approximately tripled the initial extraction rate, evidencing a marked thermal intensification of the process and supporting the selection of 5 min as a conservative operational time point for further characterization. The extracts obtained at 5 min were further characterized in terms of their phytochemical and biological profile. A two-tailed Welch’s t-test (Table A5, Appendix A) confirmed that raising the extraction temperature from 20 to 60 °C significantly (p<0.05) increased TPC, total extraction yield, selectivity, ferric-reducing antioxidant power (3.4-fold) and total anthocyanin content (1.9-fold), whereas the DPPH radical-scavenging capacity decreased significantly. This decoupling between bulk phenolic recovery and the two complementary antioxidant assays indicates that the temperature shift modifies the qualitative profile of the recovered phenolic fraction, in line with the HPLC-DAD analysis in which five glycosylated anthocyanins were identified, with petunidin-3-glucoside markedly enriched at 60 °C and peonidin-3-galactoside no longer detected at the higher temperature. Both extracts exhibited inhibitory activity against *Staphylococcus aureus* but were inactive against *Escherichia coli*, and Welch’s t-test (Table A6, Appendix A) showed no statistically significant difference between extracts obtained at 20 and 60 °C in either diffusion method (p>0.05). Under the conditions tested, this is consistent with the view that antimicrobial efficacy is not driven primarily by total phenolic content, confirming that the compound-specific basis of this activity requires fractionation, broader strain coverage and MIC determination through color-compatible readouts. Overall, these results demonstrate that conventional hydroethanolic extraction is a viable, scalable and food-grade route for the valorization of blueberry pomace as a source of bioactive phenolic compounds. Optimizing the process only by bulk phenolic indicators may, however, mask important compositional shifts that determine the functional quality of the extract. Future work should address the fractionation and compound-specific bioactivity of the recovered phenolics, as well as their incorporation and stability in real food matrices. It should be emphasized that the present study addresses the chemical recovery and the in vitro antioxidant profile of the extracts; establishing their applicability as functional ingredients will require further work on cell-based functional assays, oral bioavailability, food-matrix interactions and shelf-life under realistic processing and storage conditions, which fall beyond the scope of the current contribution.

## Figures and Tables

**Table 1:** Factor levels used in experimental design.

Feature	Levels		
	−1	0	1
$X_{1}=$ Time (min)	20	40	60
$X_{2}=$ Temperature (°C)	20	40	60
$X_{3}=$ Ethanol Concentration (%)	20	50	80

**Table 2:** Experimental FCCCD conditions and corresponding responses expressed as mean ± standard deviation (n = 3)

Experimental Run	Time	Temp.	Ethanol	TPC	Yield	Selectivity
	min	°C	%	mg GAE/g DW	%	mg GAE/g DE
1	60	60	80	10.97 ± 0.21	23.5 ± 3.7	47.5 ± 7.37
2	60	60	20	16.13 ± 0.80	19.4 ± 3.6	85.03 ± 17.3
3	60	20	80	4.58 ± 0.37	19.2 ± 0.3	23.9 ± 1.83
4	60	20	20	7.01 ± 0.31	18.6 ± 0.5	37.77 ± 2.31
5	20	60	80	9.84 ± 0.75	18.0 ± 1.1	54.93 ± 7.38
6	20	60	20	15.5 ± 0.20	16.0 ± 3.0	98.77 ± 17.46
7	20	20	80	5.36 ± 0.44	16.9 ± 0.4	31.8 ± 3.28
8	20	20	20	3.81 ± 0.38	19.8 ± 4.0	19.9 ± 5.24
9	40	40	50	13.63 ± 0.75	20.0 ± 0.6	68.13 ± 2.2
10	40	40	80	7.42 ± 0.32	17.3 ± 0.9	42.87 ± 3.69
11	40	40	20	6.73 ± 0.29	17.4 ± 0.8	38.83 ± 3.39
12	40	60	50	19.97 ± 0.98	26.8 ± 2.4	75.2 ± 9.85
13	40	20	50	12.40 ± 0.20	28.3 ± 3.8	44.27 ± 5.04
14	60	40	50	13.37 ± 1.14	20.0 ± 2.9	68.3 ± 15.0
15	20	40	50	11.60 ± 1.04	17.3 ± 2.9	67.73 ± 7.86

**Table 3:** Kinetic parameters and goodness-of-fit metrics for extraction at optimal conditions and neighboring conditions obtained at lower temperatures.

**Peleg’s Model**						
**Temperature**	$k_{1}$ **(×10^3^)**	$k_{2}$	**RMSE**	**MAE**	**AIC**	**BIC**
°C	g mg GAE^−1^	min g mg GAE^−1^				
20	19.79	0.101	1.689	1.371	26.01	28.10
60	6.69	0.048	3.622	2.919	58.05	60.14
**First-Order Model**						
**Temperature**	k	$C_{eq}$	**RMSE**	**MAE**	**AIC**	**BIC**
°C	min^−1^	mg GAE g^−1^				
20	2.93	9.56	1.755	1.399	27.62	29.71
60	3.38	20.44	3.697	2.966	58.92	61.01

**Table 4:** Retention time, relative peak area, and concentration of anthocyanins identified in blueberry pomace extracts obtained at 20 °C and 60 °C (5 min extraction time, 50% ethanol). Concentrations are expressed as mg cyanidin-3-glucoside equivalents per 100 g of dry weight (mg Cyn-3-glu E/100 g DW) and mean ± SD (n=3).

Compound	RT (min)	Relative Area (%)		Concentration (mg Cyn-3-glu E/100 g DW)	
		20 °C	60 °C	20 °C	60 °C
Delphinidin-3-galactoside	11.4	4.9	6.6	8.01 ± 0.82	17.04 ± 1.18
Cyanidin-3-galactoside	13.6	13.1	10.5	11.85 ± 3.28	23.81 ± 2.67
Petunidin-3-glucoside	19.4	49.8	52.0	29.23 ± 5.54	96.24 ± 4.01
Peonidin-3-galactoside	21.0	n.d.	1.8	n.d.	8.59 ± 0.29
Malvidin-3-galactoside	22.9	32.2	29.1	20.94 ± 4.38	56.30 ± 1.33
Total		100	100	70.04	201.98

RT: Retention time averaged across replicates. n.d.: not detected.

**Table 5:** Inhibition zone diameters (cm) against *Staphylococcus aureus*. Values expressed as mean ± standard deviation (n = 3).

Condition	Disk Diffusion (cm)	Well Diffusion (cm)
Positive control (ampicillin)	1.861 ± 0.054	2.343 ± 0.113
Extract 20 °C	0.749 ± 0.010	1.451 ± 0.060
Extract 60 °C	0.900 ± 0.100	1.489 ± 0.042
Negative control (water)	n.d.	n.d.

n.d.: not detected.

## Data Availability

The original contributions presented in this study are included in the article. Further inquiries can be directed to the corresponding author.

## Appendix A

**Table A1:** Regression coefficients and ANOVA summary for total phenolic compounds.

Term	Estimate	Std. Error	t-Value	p-Value
Intercept	13.39	0.32	42.48	<0.001
t	0.59	0.19	3.20	0.003 *
T	3.92	0.19	21.16	<0.001 *
tT	−0.08	0.21	−0.40	0.693
C	−1.10	0.19	−5.94	<0.001 *
tC	−0.44	0.21	−2.10	0.043 *
TC	−1.24	0.21	−6.00	<0.001 *
$t^{2}$	−0.85	0.37	−2.32	0.026 *
$T^{2}$	2.85	0.37	7.80	<0.001 *
$C^{2}$	−6.26	0.37	−17.11	<0.001 *
**Source**	F **-value**	p **-value**		
Model	98.10	<0.001 *		
Lack-of-fit	12.11	<0.001 *		
$R^{2}=$ 0.962				
$R_{adj}^{2}=$ 0.952				

* Significant parameter.

**Table A2:** Regression coefficients and ANOVA summary for yield.

Term	Estimate	Std. Error	t-Value	p-Value
Intercept	21.64	0.75	28.91	<0.001
t	1.25	0.44	2.84	0.008 *
T	0.09	0.44	0.21	0.835
tT	0.98	0.49	1.99	0.054
C	0.37	0.44	0.83	0.412
tC	0.70	0.49	1.42	0.163
TC	1.04	0.49	2.12	0.041 *
$t^{2}$	−3.39	0.87	−3.91	<0.001 *
$T^{2}$	5.48	0.87	6.31	<0.001 *
$C^{2}$	−4.69	0.87	−5.40	<0.001 *
**Source**	F **-value**	p **-value**		
Model	9.94	<0.001 *		
Lack-of-fit	0.62	0.687		
$R^{2}=$ 0.719				
$R_{adj}^{2}=$ 0.646				

* Significant parameter.

**Table A3:** Regression coefficients and ANOVA summary for selectivity.

Term	Estimate	Std. Error	t-Value	p-Value
Intercept	61.87	3.27	18.92	<0.001
t	−1.06	1.92	−0.55	0.584
T	20.38	1.92	10.59	<0.001 *
tT	−3.89	2.15	−1.81	0.079
C	−7.93	1.92	−4.12	<0.001 *
tC	−2.43	2.15	−1.13	0.266
TC	−9.92	2.15	−4.61	<0.001 *
$t^{2}$	7.71	3.79	2.03	0.050 *
$T^{2}$	−0.57	3.79	−0.15	0.881
$C^{2}$	−19.45	3.79	−5.13	<0.001 *
**Source**	F **-value**	p **-value**		
Model	20.51	<0.001*		
Lack-of-fit	3.72	0.010*		
$R^{2}=$ 0.841				
$R_{adj}^{2}=$ 0.800				

* Significant parameter.

**Table A4:** One-way analysis of variance (ANOVA) comparing the validation responses obtained at the two extraction temperatures (20 and 60 °C) under the selected condition (40 min, 50% ethanol; n=5 independent replicates per temperature).

Response	Unit	20 °C	60 °C	n	df	F	p-Value
TPC	mg GAE/g DW	11.92 ± 0.75	20.40 ± 1.56	5	1.8	119.77	<0.001 ***
Total yield	%	21.22 ± 0.90	24.79 ± 2.03	5	1.8	12.95	0.0070 **
Selectivity	mg GAE/g DE	56.12 ± 1.40	82.38 ± 2.67	5	1.8	378.73	<0.001 ***

Values are expressed as mean ± standard deviation. df: degrees of freedom (between groups and within groups). A one-way ANOVA was applied independently to each response, with extraction temperature as the single factor. Significance codes: *** p < 0.001; ** p < 0.01.

**Table A5:** Welch’s two-tailed t-test comparing the response variables at 20 and 60 °C for extracts obtained at 5 min of extraction (*n* = 3 independent replicates per temperature). The Benjamini–Hochberg false discovery rate (BH–FDR) correction was applied within this family of six a priori comparisons; both raw and adjusted *p*-values (*p*_adj_) are reported, and significance codes refer to *p*_adj_.

Response	Unit	20 °C	60 °C	Welch t	df	p-Value	$p_{adj}$	Sig.
TPC	mg GAE/g DW	10.06 ± 1.43	22.15 ± 0.54	13.70	2.56	0.0018	0.0036	**
Total yield	%	16.55 ± 2.20	25.36 ± 0.89	6.43	2.64	0.0111	0.0133	*
Selectivity	mg GAE/g DE	34.96 ± 2.14	76.62 ± 1.63	26.82	3.74	<0.001	<0.001	***
FRAP	µmol TE/g DW	100.96 ± 16.32	339.45 ± 12.84	19.89	3.79	<0.001	<0.001	***
DPPH	µmol TE/g DW	51.07 ± 0.66	43.97 ± 2.02	−5.79	2.42	0.0180	0.0180	*
TAC	mgC3G/g DW	2.49 ± 0.29	4.81 ± 0.51	6.85	3.17	0.0053	0.0080	**

Values are expressed as mean ± standard deviation. A two-tailed Welch’s t-test (unequal variances) was applied independently to each response, with extraction temperature as the single factor. The Welch–Satterthwaite approximation was used to compute the degrees of freedom (non-integer values). Multiple-testing correction was performed within this family of six responses using the Benjamini–Hochberg false discovery rate (BH–FDR) procedure; significance codes refer to $p_{\mathrm{adj}}$. *** $p_{\mathrm{adj}}$ < 0.001; ** $p_{\mathrm{adj}}$ < 0.01; * $p_{\mathrm{adj}}$ < 0.05.

**Table A6:** Welch’s two-tailed t-test comparing the inhibition zone diameters against *Staphylococcus aureus* between extracts at 20 and 60 °C for the disk diffusion and well diffusion assays (*n* = 3 independent replicates per temperature). The Benjamini–Hochberg false discovery rate (BH–FDR) correction was applied within this family of two *a priori* comparisons; both raw and adjusted *p*-values (*p*_adj_) are reported.

Method	Unit	20 °C	60 °C	Welch t	df	p-Value	$p_{adj}$	Sig.
Disc diffusion	cm	0.749 ± 0.010	0.900 ± 0.100	2.61	2.04	0.1182	0.2364	n.s.
Well diffusion	cm	1.451 ± 0.060	1.489 ± 0.042	0.91	3.56	0.4210	0.4210	n.s.

Values are expressed as mean ± standard deviation. A two-tailed Welch’s t-test (unequal variances) was applied independently to each method, with extraction temperature as the single factor. The Welch–Satterthwaite approximation was used to compute the degrees of freedom. Multiple-testing correction was performed within this family of two tests using the Benjamini–Hochberg false discovery rate (BH–FDR) procedure. n.s.: not significant ($p_{\mathrm{adj}}\geq 0.05$).
